# Improvements in Empathy and Cognitive Flexibility after Court-Mandated Intervention Program in Intimate Partner Violence Perpetrators: The Role of Alcohol Abuse

**DOI:** 10.3390/ijerph13040394

**Published:** 2016-03-31

**Authors:** Ángel Romero-Martínez, Marisol Lila, Manuela Martínez, Vicente Pedrón-Rico, Luis Moya-Albiol

**Affiliations:** 1 Department of Psychobiology, University of Valencia, Valencia 46010, Spain; Manuela.Martinez@uv.es (M.M.); Luis.Moya@uv.es (L.M.-A.); 2 Department of Social Psychology, University of Valencia, Valencia 46010, Spain; Marisol.Lila@uv.es (M.L.); vicentepedronrico@gmail.com (V.P.-R.)

**Keywords:** cognitive flexibility, empathy, emotion decoding, intervention programs, intimate partner violence

## Abstract

Research assessing the effectiveness of intervention programs for intimate partner violence (IPV) perpetrators has increased considerably in recent years. However, most of it has been focused on the analysis of psychological domains, neglecting neuropsychological variables and the effects of alcohol consumption on these variables. This study evaluated potential neuropsychological changes (emotional decoding, perspective taking, emotional empathy and cognitive flexibility) and their relationship with alcohol consumption in a mandatory intervention program for IPV perpetrators, as well as how these variables affect the risk of IPV recidivism. The sample was composed of 116 individuals with high alcohol (*n* = 55; HA) and low alcohol (*n* = 61; LA) consumption according to self-report screening measures who received treatment in a IPV perpetrator intervention program developed in Valencia (Spain). IPV perpetrators with HA consumption were less accurate in decoding emotional facial signals and adopting others’ perspective, and less cognitively flexible than those with LA consumption before the IPV intervention. Further, the effectiveness of the intervention program was demonstrated, with increases being observed in cognitive empathy (emotional decoding and perspective taking) and in cognitive flexibility. Nevertheless, the HA group showed a smaller improvement in these skills and higher risk of IPV recidivism than the LA group. Moreover, improvement in these skills was related to a lower risk of IPV recidivism. The study provides guidance on the targeting of cognitive domains, which are key factors for reducing IPV recidivism.

## 1. Introduction

Intimate partner violence (IPV) is a critical issue, which at some point in their lives affects around 30% of women across different populations [1]. Given the high prevalence of IPV and the serious consequences for victims of this kind of abuse [2], extensive research has been focused on interventions for the affected women. Nevertheless, it is also necessary to analyze the perpetrators of IPV to attempt to develop effective intervention programs for them and to prevent this type of violence [3]. Specifically, it is critical to examine the effectiveness of attempts to rehabilitate IPV offenders to prevent recurrent abuse, and hence protect victims. 

The effectiveness of intervention programs for IPV perpetrators depends on generating changes in IPV perpetrators and thereby preventing violence in their current and future relationships [4]. Some meta-analyses have shown pre- and post-treatment changes in psychologically meaningful risk factors such as alcohol consumption, self-esteem, attitudes toward violence, impulsivity, anger, psychological adjustment, social support, and awareness of serious offenses [1,2,5,6,7,8,9,10]. Nevertheless, these studies concluded that intervention programs have small effect sizes and limited efficacy in reducing the rate of recidivism [6,11]. Alcohol abuse is a factor which partially explains IPV maintenance after treatment (or IPV recidivism) and IPV perpetrator treatment dropout [12,13,14,15,16,17]. This factor affects several socio-cognitive skills which underlie IPV [18,19,20]. However, previous studies have not paid much attention to such variables (assessed by neuropsychological tests) or to whether they change after IPV intervention programs. Identifying these coginitive deficits and psychological traits would facilitate the development of early cognitive training initiatives that may cognitive improvements which in turn would reduce the rate of recidivism. 

A significant percentage of IPV perpetrators abuse alcohol or have alcohol use disorder [21,22]. Chronic alcohol abuse affects many socio-cognitive skills which are critical for behavioral regulation. Specifically, individuals with alcohol use disorder have been observed to have diminished emotional decoding abilities [18,23,24], theory of mind deficits and humor processing difficulties [25]. Notably, executive functions have been the most extensively studied, mainly due to their greater vulnerability to the toxic effects of alcohol [20]. Likewise, there is some overlap between these cognitive process and mental flexibility (an executive function), which is the adoption of a subjective perspective of the other. Deficits in executive function due to alcohol abuse could partially explain deficits in empathy, especially in cognitive empathy [20]. 

An important process for empathy is emotion decoding, the ability to accurately decode/understand the emotional expression of one’s interaction partner [26]. The emotion decoding process is a primary source of information, providing contextual information for making situational attributions. Moreover, it makes it possible to adopt another individual’s perspective (perspective taking), to predict others’ behavior and to estimate how to react to it. Conversely, failures in this process may lead to social inadequacy and even cause the adoption of inappropriate behavior that predisposes the individual to violent reactions [27]. Several studies have demonstrated the presence of deficits in emotional decoding in a considerable number of IPV perpetrators [20,28], which partially explains why they showed diminished perspective taking [18] and why they tend to react with violence when they find themselves in an ambiguous social situation. Furthermore, IPV perpetrators experience higher levels of personal distress (proto-emotional empathy), possibly because of difficulties recognizing affective cues from conspecifics, which is crucial in inducing affective empathy, and they tend to misunderstand how they are evaluated by others, which may also be related to difficulties in recognizing some emotional expressions [18,19,29,30,31]. However, as far as we know, there is a gap in the literature analyzing whether these deficits are temporally stable or change after IPV intervention programs. 

Executive functions are high-level cognitive functions that are involved in planning, initiation, regulation and management of behavior [32]. Therefore, alterations in executive functions may decrease behavioral control and lead to the adoption of risky behaviors [33]. This poor behavioral regulation would, in turn, reinforce the desire for immediate gratification, regardless of associated positive or negative future consequences. Thus, deficits in executive functions often lead to a failure to use available information to predict the consequences of a behavior [34]. To date, research studies have described lower levels of cognitive flexibility in IPV perpetrators as assessed by the Wisconsin Card Sorting Test (WCST); they made more errors and completed fewer categories than controls [35]. Subsequent studies replicated and expanded on these results, noting that abusers made more perseverative errors (e.g., maintained a classification criterion despite being informed that it was wrong) [19,36]. Although there is general agreement on the presence of lower cognitive flexibility among IPV perpetrators, studies to date have been cross-sectional; hence, they have not assessed whether these deficits are stable after IPV perpetrators have been treated in intervention programs.

With all this in mind, the main aim of this study was to compare empathic abilities (emotional decoding, perspective taking and emotional empathy) and executive skills in IPV perpetrators with high alcohol (HA) and low alcohol (LA) consumption before treatment in a court-mandated program for male abusers seeking to reduce the future risk of IPV. As it was previously found that IPV perpetrators with HA consumption showed poorer socio-cognitive skills (cognitive empathy and cognitive flexibility) than those with LA consumption [18], we expected to replicate those results before the IPV intervention program. Second, we also analyzed the effects of the IPV intervention program on these variables (empathy and executive functions) comparing the results before and after intervention in the sample of IPV perpetrators with HA and LA consumption. Due to the effectiveness of another intervention program in generating changes in these cognitive domains, specifically in empathy [37] and the effects of alcohol on these socio-cognitive skills and IPV maintenance [12,14,15,16,17,20,22], we hypothesized that the IPV perpetrators, especially those with LA consumption, would experience improvements in their empathic abilities (emotion decoding, perspective taking and personal distress) and executive functions (cognitive flexibility) after the intervention program. Moreover, we expected that the IPV perpetrators with LA consumption would have a lower risk of IPV recidivism than those with HA consumption. Finally, as empathy and cognitive flexibility play an important role in behavior regulation [27,38,39], we hypothesized that an improvement in these socio-cognitive skills after the treatment would be associated with a reduced risk of IPV recidivism.

## 2. Method

### 2.1. Participants

The final sample was composed of 116 IPV perpetrators. Eighty IPV perpetrators were excluded from the analysis because they did not complete the second neuropsychological assessment or did not finish the intervention. The participants were volunteers recruited from among men taking part in the *Contexto* psycho-educational and community-based treatment program (mandatory for male abusers). They had been sentenced to less than 2 years in prison and had no previous criminal record, and hence had had their sentence suspended on condition that they attended an intervention program [40,41,42,43,44]. All subjects gave their informed consent for inclusion before they participated in the study. The study was conducted in accordance with the Declaration of Helsinki, and the protocol was approved by the Ethics Committee of the University of Valencia Ethics Committee (H1348835571691).

### 2.2. The Contexto Program

The program is a community-based intervention program for IPV offenders, run at the Department of Social Psychology, University of Valencia [41]. It is based on the ecological model framework [45], recommended by the World Health Organization (WHO) [46]. The main objective of the program is to reduce risk factors and increase protective factors for IPV, taking into account four levels of analysis: individual, interpersonal, situational, and macrosocial [44,47,48]. The program begins with an *evaluation phase*, which includes the administration of several standardized tests and self-report measures, and three in-depth interviews. The main objectives in the evaluation phase are: to collect information socio-demographic characteristics, to confirm eligibility to participate in the program, and to increase motivation to complete in the program. The *intervention phase* consists of seven modules delivered over 30 weekly group sessions lasting 2 h each. It is a long group intervention, and it complies with the standards recommended in previous meta-analyses (e.g., [6]). The groups consist of 10–12 participants and are closed (no new members being enrolled after the program starts). Two professionals conduct each group. Throughout the seven modules, several intervention techniques are used, including group dynamics, presentation of contents and key concepts, group inquiry/debate, monitored exercises, case studies, role-play, videos, homework, and training on psychological strategies and techniques (e.g., cognitive restructuring, emotion management skills). In the first module, the priority is to build a climate of trust within the group and to set the norms for the working of the group. In this module, participants share experiences with the rest of the group members, describing the events that lead to their conviction. In the second module, basic concepts are explained (the meaning of partner violence, types of violence, risk factors, *etc.*). Legal terms and concepts related to their legal situation are introduced and explained. This module also introduces some activities targeting participants’ cognitive distortions and self-justifications for their situation (e.g., denial, minimization, victim-blaming) and the assumption of responsibility for their own behavior. From the third module to the sixth, the sessions aim to increase resources and skills, as well as to reduce risk factors at the individual level (third module: e.g., emotional control techniques, self-concept, and self-esteem), interpersonal level (fourth module; e.g., positive communication skills in intimate relationships, awareness of the impact of IPV on children), situational level (fifth module: e.g., social integration and support), and sociocultural level (sixth module: e.g., gender roles and sexist attitudes, co-domesticity). In the seventh module, sessions focus on recidivism prevention and consolidating learning and training objectives. The *follow-up phase* lasts 18 months starting from the end of the program sessions, with six follow-up sessions held every 3 months.

### 2.3. Procedure

Each participant in the study attended five sessions (evaluation phase) in the psychology laboratories of the University of Valencia. In the first session, they were interviewed to exclude any individuals with organic diseases. The second session took place the day after the first and was completed between 10 a.m. and 2 p.m. in order to minimize possible effects of fatigue later in the day. After arriving at the laboratory, participants were taken to a room where they signed an informed consent form to participate in the study, and data were collected on demographic and anthropometric variables (age), alcohol consumption and social desirability. Then, two neuropsychological tests were administered, the WCST and the Reading the Mind in the Eyes (Eyes Test). Finally, in a third session, participants completed various empathy evaluation questionnaires. After the intervention program, the second and third sessions were repeated (one week before the end of the intervention phase), assessing the same neuropsychological and psychological variables.

### 2.4. Alcohol Abuse Evaluation and Social Desirability

The Spanish version of the Alcohol Use Disorders Identification Test (AUDIT) [49] incorporates questions about the quantity and frequency of alcohol consumption in adults. It has been developed by the WHO to identify persons whose alcohol consumption has become hazardous or harmful to their health. It is composed of 10 self-report items ranging from 0 (never) to 4 (daily or almost daily). All the response scores should then be added in a total score that ranged from 0 to 40. Total scores of 8 or more are recommended as indicators of hazardous and harmful alcohol use, as well as possible alcohol dependence. The AUDIT is distinguished from other well-known screening tools in that items are scored on a frequency continuum (rather than dichotomously), it focuses on a limited time period (e.g., 6 months *vs.* lifetime), and it appears to have broader applicability by detecting hazardous and harmful drinkers (*i.e.*, at-risk problem drinkers) rather than those who are alcohol dependent [50]. The Cronbach’s alpha was 0.88.

The Millon Clinical Multiaxial Inventory-III (MCMI-III) [51] self-report inventory consisting of 175 dichotomous items which measure personality disorders was also used. It comprises 11 Clinical Personality Patterns scales, three Severe Personality scales, seven Clinical Syndromes scales, three Severe Syndrome scales, and three Modifying scales. For this study, in addition to the Desirability scale (mentioned below), the Alcohol use disorder scale was used to assess alcohol use. A reliability of between 0.65 and 0.92 was found in the validation of the Spanish version. Social desirability was measured using the Desirability scale of the aforementioned MCMI-III.

### 2.5. Empathy

The Interpersonal Reactivity Index (IRI) assesses four aspects of empathic response [52], and for this study, we used the Spanish adaptation [53]. The instrument is composed of four subscales (perspective taking, fantasy, empathic concern, and personal distress) ranked on a 5-point Likert scale with reliability coefficients ranging from 0.56 to 0.70. 

The revised version of the Eyes Test was also administered [54]. Considered an advanced theory of mind test, it contains 36 black and white photographs of the eye region of the face of different male and female actors, and participants are asked to assess the mental state of the people in the photographs. Specifically, participants were instructed to choose which of four words best described what the person in each photo was thinking or feeling. Scores are calculated as the total number of correct choices for all 36 photographs. The Cronbach’s alpha was 0.79.

### 2.6. Cognitive Flexibility

The revised version of the WCST [55] was used to measure cognitive flexibility. Cards must be sorted until six categories have been matched or until all 128 cards have been sorted. Participants were seated in front of four cards, and were told to match the card on the bottom of the table with one of the four cards in the top of the table. Cards are matched according to various criteria, such as color, form, and number. They were not told how to match the cards, but feedback (correct/wrong) was given after each trial. In each trial, only one sorting criterion was correct, and such criterion changed every 10 trials, thus measuring adaptation to changing rules. Once 10 consecutive cards were correctly sorted, a new criterion was introduced without warning the participants. We assessed the number of trials, the number of categories completed, number of perseverative errors (number of times in which the participant persisted in responding to an incorrect stimulus characteristic) and the percentage of perseverative errors (number of times in which the participant persisted in responding to an incorrect stimulus characteristic over the total number of responses).

### 2.7. Risk of Recividism

Spousal Assault Risk Assessment Guide (SARA) [56], which was completed by trained evaluators [57], includes a set of 20 risk factors for spousal violence on a 3-point scale (0 = low to 2 = high risk). The risk factors are related to risk of violence in general and risk of spousal violence in particular. Sample items included “recent relationship problems”, “recent psychotic and/or manic symptoms” and “personality disorder with anger, impulsivity, or behavioral instability”. Evaluators have to code the presence of each risk factor, whether any are considered “critical”, and the overall degree of risk posed by the participant. For this study, we considered the sum of the risk factors.

### 2.8. Data Analysis

Cluster analysis includes a variety of multivariate statistical procedures used to classify individuals into relatively homogeneous groups [58]. In this study, K-means cluster analyses were conducted to determine the subgroups, focusing on the following measures: (a) the AUDIT score; and (b) the score on the Alcohol Use Disorder scale from the MCMI-III. These analyses resulted in the formation of two groups. Ninety-two and 89 IPV perpetrators were grouped as having HA, that is, those with higher than average scores on AUDIT and Alcohol Use Disorder MCMI-III, and LA consumption, that is, those participants with lower scores than average on the AUDIT and the MCMI-III. The HA and LA scores were 8.49 ± 1.55 and 3.36 ± 2.33 for the AUDIT, respectively, and 69.02 ± 9.77 and 25.95 ± 6.99 for the Alcohol Use Disorder MCMI-III, respectively.

It was previously established using the Kolmogorov-Smirnov statistic (*p* < 0.001) that the data were normally distributed. *T*-tests were carried out to check significant differences between groups (IPV perpetrators with HA and LA consumption) in anthropometric variables, social desirability, empathy, cognitive flexibility and sexism before the intervention program. Further, *t*-tests were also employed to compare SARA scores after the intervention program. Effect sizes for the between-group differences were calculated using Cohen’s d [59]. Chi square analyses were performed for demographic variables, presence or absence of physical IPV before intervention program and rates of recidivism after the intervention program.

The effectiveness of the intervention program was confirmed by repeated-measures ANOVA (general linear model) with “time” (pre and post) as a within-participant factor. To examine group effects, repeated-measures ANOVA was conducted with “time” as the within-participant and “group” as the between-participant factors. Greenhouse–Geisser corrections for degrees of freedom and Bonferroni corrections for multiple comparisons were applied where appropriate. For significant results, partial eta squared (ηp^2^) is reported as a measure of effect size. All statistical analyses were performed with SPSS 22.0 for Windows (IBM Corperation, Armonk, NY, USA) with the alpha level fixed at 0.05.

## 3. Results

Descriptive characteristics, psychological trait profiles and neuropsychological variables for the IPV perpetrators with HA and LA are summarized in Table 1. Groups did not differ in age, nationality, economic status and educational level. Nonetheless, there were significant differences between groups in marital status, χ^2^(4) = 17.19, *p* = 0.007, with more individuals being single in the HA than the LA group. Moreover, there were differences between groups in presence of physical IPV before the implementation of Intervention program χ^2^(1) = 4.82, *p* = 0.028, with more individuals of the HA group being reported for evidence of physical IPV than in IPV perpetrators of LA group. Finally, a significant “group” effect was found in MCMI-III Desirability scores, t(114) = −2.74, *p* = 0.000, *d* = 0.51, with lower social desirability scores being obtained in the HA than the LA group.

### 3.1. Before Intervention Program

#### 3.1.1. Empathy

Regarding empathy, IPV perpetrators with HA consumption obtained lower perspective taking and Eyes Test scores than did those with LA consumption, t(114) = −2.06, *p* = 0.046, *d* = 0.39, and t(114) = −1.88, *p* = 0.053, *d* = 0.35, respectively. Additionally, the HA group obtained higher scores on the personal distress scale, t(114) = 2.10, *p* = 0.040, *d* = 0.37, than the LA group. Nevertheless, they did not differ in fantasy or empathic concern, t(114) = −1.05, *p* = 0.297 and t(114) = −0.383, *p* = 0.702, respectively.

#### 3.1.2. Cognitive Flexibility

Regarding performance on the WCST, IPV perpetrators with HA consumption completed fewer categories, t(114) = −2.10, *p* = 0.038, *d* = 0.39, committed more perseverative errors, t(114) = 2.66, *p* = 0.009, *d* = 0.50, and obtained a higher percentage of perseverative errors, t(114) = −2.10, *p* = 0.038, *d* = 0.39 than those with LA consumption. Additionally, they used more trials to complete the categories, t(114) = 3.14, *p* = 0.002, *d* = 0.59, than those in the LA group. 

#### 3.1.3. Risk of Recidivism before Intervention Program (SARA)

With regard to the risk of recidivism before the implementation of the intervention program, IPV perpetrators with HA showed a higher risk of recidivism, t(114) = 2.64, *p* = 0.010, *d* = 0.49 than those with LA consumption. 

### 3.2. Effectiveness of the Intervention Program to Elicit Empathy, and Strengthen Cognitive Flexibility 

Effectiveness of the Intervention Program for the IPV perpetrators with HA and LA is presented in Table 2.

#### 3.2.1. Empathy

A significant effect of “time” being found in the total sample on the Eyes Test and perspective taking scores, F(1, 89) = 4.46, *p* = 0.037 ηp^2^ = 0.05 and F(1, 114) = 6.52, *p* = 0.012, ηp^2^ = 0.05, respectively. However, there were no find significant “time” effects in IRI personal distress, fantasy or empathic concern. After analyzing each group separately, within-group comparisons revealed significant effects for “time” in the Eyes Test in both groups, IPV perpetrators with HA consumption, F(4, 68) = 6.14, *p* = 0.001, ηp^2^ = 0.27, and those with LA consumption, F(1, 43) = 7.95, *p* = 0.007, ηp^2^ = 0.16; F(1, 60) = 7.98, *p* = 0.006; ηp^2^ = 0.12. Nevertheless, “time” was only significant for perspective taking in the LA group, F(1, 43) = 7.95, *p* = 0.007, ηp^2^ = 0.16. Regarding the other three IRI subscales, no significant “time” effects were found. The two groups followed a similar pattern, with an increase in empathy scores after the intervention program. Notably, however, a significant “time × group” interaction was observed in the Eyes Test and perspective taking, F(1, 89) = 4.90, *p* = 0.029, ηp^2^ = 0.05; F(1, 114) = 3.68, *p* = 0.050, ηp^2^ = 0.04, with the HA group presenting lower scores at both assessment points. After including “social desirability” as a covariate the “time x group” interactions were still significant for Eyes test and perspective taking (F(1, 88) = 4.03, *p* = 0.048, ηp^2^ = 0.04; F(1, 113) = 3.72, *p* = 0.050, ηp^2^ = 0.03, respectively).

#### 3.2.2. Cognitive Flexibility

In the WCST, a significant “time” effect was found in the total sample for number of trials to complete the categories, number of categories, and number and percentage of perseverative errors, F(1, 114) = 11.03, *p* = 0.001; ηp^2^ = 0.11; F(1, 114) = 8.05, *p* = 0.006; ηp^2^ = 0.08; F(1, 114) = 34.57, *p* = 0.000, ηp^2^ = 0.27, and F(1, 114) = 10.94, *p* = 0.000, ηp^2^ = 0.10, respectively. Dividing the sample into groups, intra-group comparisons revealed a significant “time” effect in the HA group, F(1, 53) = 4.85, *p* = 0.033, ηp^2^ = 0.10; F(1, 59) = 3.50, *p* = 0.060, ηp^2^ = 0.08, and the LA group, F(1, 53) = 3.42, *p* = 0.060, ηp^2^ = 0.07; F(1, 59) = 49.72, *p* = 0.000, ηp^2^ = 0.50. In both groups, WCST performance improved after the intervention program, with increases in the number of categories completed and decreases in the number and percentage of perseverative errors and number of trials. Further, a significant “time × group” effect was found in number of categories completed and perseverative errors, F(1, 114) = 10.19, *p* = 0.002, ηp^2^ = 0.10; F(1, 114) = 8.26, *p* = 0.005, ηp^2^ = 0.08, respectively, with the HA group completing fewer categories and making more perseverative errors after the intervention program than the LA group. After including “social desirability” as a covariate the “time × group” interactions were still significant for number of categories completed and perseverative errors (F(1, 113) = 8.12, *p* = 0.005, ηp^2^ = 0.08; F(1, 114) = 3.56, *p* = 0.050, ηp^2^ = 0.04, respectively).

#### 3.2.3. Risk of Recidivism (SARA) and Recidivism

A significant “time” effect was found in the total sample for the risk of recidivism, F(1, 114) = 82.36, *p* = 0.001; ηp^2^ = 0.43. Dividing the sample into groups, intra-group comparisons revealed a significant “time” effect in the HA group, F(1, 53) = 39.25, *p* = 0.001, ηp^2^ = 0.45, and the LA group, F(1, 59) = 42.91, *p* = 0.001, ηp^2^ = 0.43. In both groups the risk of recidivism decreased after the finalization of the intervention program. In addition, a significant “group” effect was found in SARA scores, F(1, 112) = 7.37, *p* = 0.008, ηp^2^ = 0.06, with the HA group presenting a higher risk of recidivism than the LA group (8.63 ± 3.92 and 6.7 ± 3.44, respectively). After including “social desirability” as a covariate the “group” effect was still significant for the risk of recidivism (F(1, 111) = 4.81, *p* = 0.030, ηp^2^ = 0.04). Finally, a significant “group” effect was found in the rates of recidivism χ²(1) = 6.73, *p* = 0.009, with the HA group having more cases of recidivism after the intervention program than the LA group.

### 3.3. Relationship of Empathy and Cognitive Flexibility Variables Assessed after Intervention Program with Risk of Recidivism 

Risk of recidivism was significantly and negatively associated with IRI perspective taking and Eyes Test scores (*r* = −0.205 and *r* = −0.301, *p* < 0.01, respectively). Furthermore, it was significantly and positively associated with the number and percentage of perseverative errors (*r* = 0.373 and *r* = 0.390, *p* < 0.001, respectively). However, it was unrelated to the IRI personal distress, empathic concern and fantasy scores and WCST number of categories and trials completed. After dividing the sample (HA *vs.* LA), similar correlation coefficients were obtained for both groups, indicating the same pattern of relationships.

## 4. Discussion

The present study has demonstrated that IPV perpetrators with HA consumption were less accurate in decoding emotional facial signals and adopting others’ perspective and less cognitively flexible than those with LA before the IPV intervention. Further, they showed higher personal distress than the LA group. Moreover, we found that the HA group showed a smaller improvement in these skills than the LA group, who also presented a lower risk of IPV recidivism after the intervention program. Finally, as expected, improvement in these socio-cognitive skills was associated with a lower risk of IPV recidivism or maintenance.

The first main objective of the current study was to assess differences in empathy (cognitive and emotional) and cognitive flexibility in two groups with different alcohol consumption levels (high *vs.* low or abstinence). It is likely that, at least in a considerable number of cases, chronic consumption of alcohol plays a functional role in the occurrence of IPV [15], underlying the executive functions and empathic dysfunctions which facilitate this kind of violence [18,20]. Our findings support this hypothesis in that the IPV perpetrators with HA consumption obtained lower Eyes Test, perspective taking and cognitive flexibility scores than those with LA consumption. Our results describe the IPV perpetrators with HA consumption as a group of people with a reduced capacity to process information. Moreover, it seems that cognitive empathy (emotional recognition and perspective taking) is more impaired than emotional empathy in this kind of IPV perpetrators. The model for explaining the facilitation of the violence under the influence of alcohol is the *Myopic Model*, which states that alcoholic intoxication worsens attention capacities and/or information processing. In turn, this toxicity tends to restrict the perception of external and internal information and the focusing of conscious perception on a small number of salient stimuli, neglecting other information, increases the likelihood of a violent reaction [60]. 

A second main objective of the current study was to examine the effectiveness of the IPV intervention program in generating changes in empathic abilities and cognitive flexibility. Firstly, our results demonstrated that emotional decoding abilities, perspective taking and cognitive flexibility improved after the intervention program. As the Eyes Test and the WCST showed a high test-retest reliability and stability over a 1-year period [61,62], it is highly probable that the changes observed in these tests in our study are attributable to the intervention program, rather than limitations of the tests used. Further, all the IPV perpetrators also experienced an improvement in their perspective taking and a reduction in their personal distress (or negative self-oriented feelings). In relation to this, an improvement in the recognition of emotional expressions could help to increase accuracy in recognizing others’ feelings and thoughts and inducing affective empathy [63]. Regarding the cognitive rigidity or poor cognitive flexibility which are closely related to sexist beliefs [18], it makes sense that the training in cognitive restructuring and emotion management skills provided in our intervention program would improve cognitive flexibility, in line with the significant reduction in sexist beliefs (perseverative cognitive schemas) observed after a IPV perpetrator intervention program in another sample of IPV perpetrators [64]. Finally, it is well established that alcohol interferes in IPV treatment, increasing the risk of IPV recidivism [12,14,15,16,17,22]. Our data is congruent with this in that the IPV perpetrators with HA consumption showed smaller improvements in socio-cognitive skills than those with LA consumption, in some cases showing no improvement at all. Hence, these results reinforce the idea that it is necessary to pay more attention to alcohol abuse in IPV perpetrators, and include additional strategies such as drug abuse therapies, because the associated toxicity diminishes their cognitive skills, reducing the potential benefits from IPV intervention programs.

Although the correlational nature of our data means that causal relationships cannot be established linking the empathy processes and cognitive flexibility with IPV recidivism, the pattern of findings obtained in our study is consistent with the hypothesis that difficulties in recognizing some emotional expressions and adopting others’ perspective and presenting high cognitive rigidity might be involved in impairments in the regulation of behavior and the adoption of inappropriate behaviors such as IPV [20,27,28]. Notably, we found the same pattern of relationships between these variables in both groups.

This is the first study to examine the effectiveness of an intervention for male abusers assessing changes in specific socio-cognitive domains (empathy and cognitive flexibility) after the intervention program. A strength of our study is that we report these findings in a relatively large sample of IPV perpetrators. Moreover, we strengthen our findings with neuropsychological tests, which do not present the limitations of psychological assessments. On the other hand, the main limitation of this study is the absence of a control group, which would have helped to confirm that the observed changes were caused by the intervention and not by uncontrolled variables. Furthermore, IPV recidivism was only assessed by the program staff, instead of considering this assessment together with partner reports. Moreover, the current study may not be able to account for the specific component of the intervention which was the agent of change for the specific variables analyzed in our study. Future studies will benefit from larger and more structured assessment of different cognitive domains with a neuropsychological battery and, additionally, analyzing all of the variables in controls with and without a history of violence and alcohol abuse. Moreover, it could be interesting to study these neuropsychological changes in different IPV perpetrator intervention modalities or programs. 

## 5. Conclusions

In conclusion, this study shows that the IPV intervention program for IPV perpetrators studied improved several types of socio-cognitive skills in IPV perpetrators, these improvements being larger in IPV perpetrators with lower alcohol consumption. Moreover, our study supports the view that alcohol is a key factor limiting the effectiveness of IPV intervention programs, interfering in cognitive changes after the intervention and increasing the risk of recidivism. Further, our study has enabled us to explore the cognitive deficits underlying IPV perpetration, these being key factors for reducing the risk of IPV recidivism.

## Figures and Tables

**Table 1:** Mean + SD of descriptive characteristics, psychological trait profiles and neuropsychological variables for High Alcohol (HA) and Low Alcohol (LA) IPV perpetrators.

Descriptive Characteristics		High Alcohol (*n* = 55)	Low Alcohol (*n* = 61)
Age (years)		39.59 ± 9.70	42.21 ± 11.22
Educational level	Basics	9%	5%
	Graduate	57%	50%
	College	34%	45%
Nationality	Spanish	75%	78%
	Latin Americans	16%	7%
	Africans	5%	7%
	Eastern Europe Countries	4%	8%
Employment status	Working full or part time	50%	58%
	Unemployed	50%	42%
Economic income per year	<1800 €	23%	15%
	1800–12,000 €	50%	46%
	12,000–36,000 €	23%	38%
	>36,000 €	0%	1%
Marital status **	Single	47%	25%
	Married	26%	28%
	Divorced	28%	47%
Presence of physical IPV before Implementation of Intervention program *	Yes No	87% 13%	70% 30%

* *p* < 0.05; ** *p* < 0.01.

**Table 2:** Mean ± SD for empathy variables and WCST (pre vs post-treatment) for High Alcohol (HA) and Low Alcohol (LA) IPV perpetrators.

Effectiveness of the Intervention Program		Pre-Treatment		Post-Treatment	
		High Alcohol	Low Alcohol	High Alcohol	Low Alcohol
Eyes test *		16.78 ± 6.18	19.59 ± 4.29	18.11 ± 5.88	22.58 ± 6.62
**IRI**	Perspective taking *	18.91 ± 6.98	21.15 ± 4.61	20.15 ± 5.50	24.87 ± 7.27
	**Personal distress**	14.91 ± 4.14	12.92 ± 3.30	13.96 ± 4.11	13.18 ± 3.77
	**Fantasy**	16.22 ± 5.42	15.11 ± 4.75	15.75 ± 5.49	15.31 ± 4.86
	**Empathic concern**	24.73 ± 4.56	25.36 ± 4.60	24.20 ± 4.07	24.40 ± 4.28
**WCST**	**Number of trials**	120.73 ± 16.22	116.20 ± 19.83	110.04 ± 22.00	105.70 ± 25.08
	Number of categories completed **	3.07 ± 2.24	4.10 ± 2.24	3.22 ± 2.08	5.04 ± 1.98
	Number of perseverative errors **	29.21 ± 19.79	22.09 ± 12.24	29.84 ± 19.87	18.12 ± 11.81
	**Percentage of perseverative errors**	23.47 ± 15.33	18.00 ± 12.03	23.43 ± 13.97	17.38 ± 9.65
SARA (risk of recidivism) **		10.26 ± 4.48	8.08 ± 4.35	7.09 ± 4.18	5.36 ± 3.08
Rates of Recidivism *	**Yes** **No**			15% 85%	2% 98%

* *p* < 0.05; ** *p* < 0.01.

## References

[1] World Health Organization (WHO) (2013). Global and Regional Estimates of Violence against Women: Prevalence and Health Effects of Intimate Partner Violence and Non-Partner Sexual Violence.

[2] Eckhardt C.I., Murphy C.M., Whitaker D.J., Sprunger J., Dykstra R., Woodard K. (2013). The effectiveness of intervention programs for perpetrators and victims of intimate partner violence. Partner Abuse.

[3] Pinto L.A., Sullivan E.L., Ronsebaum A., Wyngarden N., Umhau J.C., Miller M.W., Taft C.T. (2010). Biological correlates of intimate partner violence perpetration. Aggress. Violent Behav..

[4] Lila M., Oliver A., Catalá-Miñana A., Galiana L., Gracia E. (2014). The Intimate Partner Violence Responsibility Attribution Scale (IPVRAS). Eur. J. Psychol. Appl. Legal Context.

[5] Arias E., Arce R., Vilariño M. (2013). Batterer intervention programmes: A metaanalytic review of effectiveness. Psychosoc. Interv..

[6] Babcock J.C., Green C.E., Robie C. (2004). Does batterer’s treatment work? A metaanalytic review of domestic violence treatment. Clin. Psychol. Rev..

[7] Davis R.C., Taylor B.G. (1999). Does batterer treatment reduce violence? A synthesis of the literature. Women Crim. Justice.

[8] Feder L., Wilson D. (2005). A meta-analytic review of court-mandated batterer intervention programs: Can courts affect abusers’ behavior?. J. Exp. Criminol..

[9] Norlander B., Eckhardt C. (2005). Anger, hostility, and male perpetrators of intímate partner violence: A meta-analytic review. Clin. Psychol. Rev..

[10] Smedslund G., Dalsbø T.K., Steiro A.K., Winsvold A., Clench-Aas J. (2011). Cognitive behavioural therapy for men who physically abuse their female partner. Cochrane Database Syst. Rev..

[11] Aldarondo E., Mederos F. (2002). Programs for Men Who Batter: Intervention and Prevention Strategies in a Diverse Society.

[12] Boira S., Jodrá P. (2010). Psicopatología, características de la violencia y abandonos en programas para hombres violentos con la pareja: Resultados en un dispositivo de intervención. Psicothema.

[13] Catalá-Miñana A., Lila M., Conchell R., Romero-Martínez A., Moya-Albiol L. (2013). ¿Se benefician de los programas de intervención que no tratan específicamente el consumo de alcohol los maltratadores con problemas de consumo abusivo?. Psychosoc. Interv..

[14] Dalton B. (2001). Batterer characteristics and treatment completion. J. Interpers. Violence.

[15] Klostermann K.C., Fals-Stewart W. (2006). Intimate partner violence and alcohol use: Exploring the role of drinking in partner violence and its implications for intervention. Aggress. Violent Behav..

[16] Stuart G.L. (2005). Improving violence intervention outcomes by integrating alcohol treatment. J. Interpers. Violence.

[17] Tollefson D.R., Gross E.R. (2006). Predicting recidivism following participation in a treatment program for batterers. J. Social Serv. Res..

[18] Romero-Martínez A., Lila M., Catalá-Miñana A., Williams R.K., Moya-Albiol L. (2013). The contribution of childhood parental rejection and early androgen exposure to impairments in socio-cognitive skills in intimate partner violence perpetrators with high alcohol consumption. Int. J. Environ. Res. Public Health.

[19] Romero-Martínez A., Lila M., Sariñana-González P., González-Bono E., Moya-Albiol L. (2013). High testosterone levels and sensitivity to acute stress in perpetrators of domestic violence with low cognitive flexibility and impairments in their emotional decoding process: A preliminary study. Aggressive Behavior..

[20] Romero-Martínez A., Moya-Albiol L. (2013). Neuropsychology of perpetrators of domestic violence: The role of traumatic brain injury and alcohol abuse and/or dependence. Rev. Neurol..

[21] Stuart G.L., O´Farrell T.J., Temple J.R. (2009). Review of the association between treatment for substance misuse and reductions in intimate partner violence. Subst. Use Misuse..

[22] Catalá-Miñana A., Lila M., Oliver A. (2013). Consumo de alcohol en hombres penados por violencia contra la pareja: Factores individuales y contextuales. Adicciones.

[23] Maurage P., Grynberg D., Noël X., Joassin F., Philippot P., Hanak C., Verbanck P., Luminet O., de Timary P., Campanella S. (2011). Dissociation between affective and cognitive empathy in alcoholism: A specific deficit for the emotional dimension. Alcohol Clin. Exp. Res..

[24] Thoma P., Friedmann C., Suchan B. (2013). Empathy and social problem solving in alcohol dependence, mood disorders and selected personality disorders. Neurosci. Biobehav. Rev..

[25] Uekermann J., Daum I. (2008). Social cognition in alcoholism: A link to prefrontal cortex dysfunction?. Addiction.

[26] Gery I., Miljkovitch R., Berthoz S., Soussignan R. (2009). Empathy and recognition of facial expressions of emotion in sex offenders, non-sex offenders and normal controls. Psychiatry Res..

[27] Tirapu-Ustárroz J., Pérez-Sayes G., Erekatxo-Bilbao M., Pelegrín-Valero C. (2007). What is theory of mind?. Rev. Neurol..

[28] Babcock J.C., Green C.E., Webb S.A. (2008). Decoding deficits of different types of batterers during presentation of facial affect slides. J. Fam. Viol..

[29] Moriguchi Y., Decety J., Ohnishi T., Maeda M., Mori T., Nemoto K., Matsuda H., Komaki G. (2007). Empathy and judging other’s pain: An fMRI study of alexithymia. Cereb. Cortex.

[30] Rogers K., Dziobek I., Hassenstab J., Wolf O.T., Convit A. (2007). Who cares? Revisiting empathy in Asperger syndrome. J. Autism Dev. Dis..

[31] Smith M.J., Horan W.P., Karpouzian T.M., Abram S.V., Cobia D.J., Csernansky J.G. (2012). Self-reported empathy deficits are uniquely associated with poor functioning in schizophrenia. Schizophr. Res..

[32] Tirapu-Ustárroz J., Muñoz-Céspedes J.M., Pelegrín-Valero C., Albéniz-Ferreras A. (2005). A proposal for a protocol for use in the evaluation of the executive functions. Rev. Neurol..

[33] Heinz A.J., Beck A., Meyer-Lindenberg A., Sterzer P., Heinz A. (2011). Cognitive and neurobiological mechanisms of alcohol-related aggression. Nat. Rev. Neurosci..

[34] Verdejo A., Orozco-Gimenez C., Meersmans-Sanchez-Jofre M., Aguilar de Arcos F., Perez-Garcia M. (2004). The impact exerted by the severity of recreational drug abuse on the different components of the executive function. Rev. Neurol..

[35] Teichner G., Golden C.J., Van Hasselt V.B., Peterson A. (2001). Assessment of cognitive functioning in men who batter. Int. J. Neurosci..

[36] Easton C.J., Sacco K.A., Neavins T.M., Wupperman P., George T.P. (2008). Neurocognitive performance among alcohol dependent men with and without physical violence toward their partners: A preliminary report. Am. J. Drug Alcohol Abuse.

[37] Scott K.L., Wolfe D.A. (2003). Readiness to change as a predictor of outcome in batterer treatment. J. Consult. Clin. Psychol..

[38] Moya-Albiol L. (2014). Empatía Entenderla Para Entender a Los Demás.

[39] Moya-Albiol L., Herrero N., Bernal M.C. (2010). The neural bases of empathy. Rev. Neurol..

[40] Lila M. (2013). La intervención con hombres condenados por violencia de pareja contra la mujer en España: Investigación y avances en intervención. Psychosoc. Interv..

[41] Lila M., García A., Lorenzo M.V. (2010). Manual de Intervención con Maltratadores.

[42] Lila M., Gracia E., García F. (2010). Actitudes de la policía ante la intervención en casos de violencia contra la mujer en las relaciones de pareja: Influencia del sexismo y la empatía. Rev. Psicol. Soc..

[43] Lila M., Gracia E., Herrero J. (2012). Asunción de responsabilidad en hombres maltratadores: Influencia de la autoestima, la personalidad narcisista y la personalidad antisocial. Rev. Latinoam. Psicol..

[44] Lila M., Gracia E., Murgui S. (2013). Psychological adjustment and victim-blaming among intimate partner violence offenders: The role of social support and stressful life events. Eur. J. Psychol. Appl. Legal Context.

[45] Heise L.L. (1998). Violence against women: An integrated, ecological framework. Violence Against Women.

[46] Dahlberg L.L., Krug E.G., Krug E.G., Dahlberg L.L., Mercy J.A., Zwi A.B., Lozano R. (2002). Violence: A global public health problem. World Report on Violence and Health.

[47] Catalá-Miñana A., Walker K., Bowen E., Lila M. (2014). Cultural differences in personality and aggressive behavior in intimate partner violence offenders: A comparison of English and Spanish offenders. J. Interpers. Violence.

[48] Gracia E., López-Quilez A., Marco M., Lladosa S., Lila M. (2014). Exploring neighborhood influences on small-area variations in intimate partner violence risk: A bayesian random-effects modeling approach. Int. J. Environ. Res. Public Health.

[49] Contell-Guillamón C., Gual-Solé A., Colom-Farran J. (1999). Test para la identificación de transtornos por uso de alcohol (AUDIT): Traducción y validación del AUDIT al catalán y castellano (in Spanish). Adicciones.

[50] Babor T.E., Grant M.G. (1989). From clinical research to secondary prevention: International collaboration in the development of the Alcohol Use Disorders Identification Test (AUDIT). Alcohol Health Res. World.

[51] Millon T., Davis R.D., Millon C. (2007). MCMI-III, Inventario Clínico Multiaxial de Millon-III.

[52] Davis M.H. (1983). Measuring individual differences in empathy: Evidence for a multidimensional approach. J. Pers. Soc. Psychol..

[53] Mestre V., Frías M.D., Samper P. (2004). La medida de la empatía: Análisis del interpersonal reactivity index (in Spanish). Psichotema.

[54] Baron-Cohen S., Wheelwright S., Hill J., Raste Y., Plumb I. (2001). The “Reading the Mind in the Eyes” Test revised version: A study with normal adults, and adults with Asperger syndrome or high-functioning autism. J. Child Psychol. Psychiatry.

[55] Heaton R.K., Chelune G.J., Talley J.L., Kay G.G., Curtis G. (2011). Test de Clasificación de Tarjetas de Wisconsin (in Spanish).

[56] Pueyo A.A., López S., Álvarez Y.E. (2008). Valoración del riesgo de violencia contra la pareja por medio de la SARA. Papeles Psicól..

[57] Kropp R., Hart S.D. (2000). The Spousal Assault Risk Assessment Guide (SARA): Reliability and validity in adult male offenders. Law Hum. Behav..

[58] Aldenderfer M.S., Blashfield R.K. (1984). Cluster Analysis.

[59] Cohen J. (1988). Statistical Power Analysis for the Behavioral Sciences.

[60] Giancola P.R., Duke A.A., Ritz K.Z. (2011). Alcohol, violence, and the alcohol myopia model: Preliminary findings and implications for prevention. Addict. Behav..

[61] Fernández-Abascal E.G., Cabello R., Fernández-Berrocal P., Baron-Cohen S. (2013). Test-retest reliability of the “Reading the Mind in the Eyes” test: A one-year follow-up study. Mol. Autism.

[62] Ingram F., Greve K.W., Ingram P.T., Soukup V.M. (1999). Temporal stability of the Wisconsin Card Sorting Test in an untreated patient sample. Br. J. Clin. Psychol..

[63] Decety J., Lamm C. (2006). Human empathy throughthe lens of social neuroscience. ScientificWorldJournal.

[64] Craig M.E., Robyak J., Torosian E.J., Hummer J. (2006). A study of male veterans beliefs toward domestic violence ina batterers intervention program. J. Interpers. Violence.

